# Clinical evaluation of large language model recommendations in melanoma: comparison with multidisciplinary tumor board decisions in a real-world cohort

**DOI:** 10.3389/fonc.2026.1856390

**Published:** 2026-06-29

**Authors:** Belma Babic, Sefika Umihanic, Hedim Osmanovic, Nejra Selak, Erna Sehic-Kozica, Lejla Moranjkic, Inga Marijanovic, Marija Karaga, Amina Jalovcic Suljevic, Sekib Umihanic, Fadil Umihanic, Arzumana Ozegovic-Orucevic

**Affiliations:** 1 Department of Pulmonary Diseases, University Clinical Center Tuzla, Tuzla, Bosnia and Herzegovina; 2 Department of Oncology and Radiotherapy, University Clinical Center Tuzla, Tuzla, Bosnia and Herzegovina; 3 Faculty of Natural Sciences and Mathematics, University of Tuzla, Tuzla, Bosnia and Herzegovina; 4 Department of Pathology, Medical Faculty University of Sarajevo, Sarajevo, Bosnia and Herzegovina; 5 Department of Oncology and Radiotherapy, Cantonal Hospital Zenica, Zenica, Bosnia and Herzegovina; 6 Department of Oncology, University Clinical Hospital Mostar, Mostar, Bosnia and Herzegovina; 7 Department of Oncology, Clinical Center University of Sarajevo, Sarajevo, Bosnia and Herzegovina; 8 Otorhinolaryngology (ENT) Department, University Clinical Center Tuzla, Tuzla, Bosnia and Herzegovina; 9 Cerrahpasa Faculty of Medicine, Istanbul University-Cerrahpasa, Istanbul, Türkiye

**Keywords:** clinical decision support, large language models, melanoma, multidisciplinary team board, oncology

## Abstract

**Background:**

Large language models (LLMs) are increasingly being studied as potentially valuable support tools in oncology practice including clinical decision support. Yet, their real-world utility in melanoma treatment decision-making is still not sufficiently considered, especially in resource-limited settings. Accordingly, this study evaluated the performance of four LLMs against real-world treatment decisions of a melanoma multidisciplinary tumor board (MDT).

**Methods:**

This retrospective single-center study included 151 consecutive patients with newly diagnosed cutaneous melanoma discussed at the MDT at the University Clinical Center Tuzla, Bosnia and Herzegovina, between 2020 and 2024. Melanoma treatment recommendations generated by four LLMs, ChatGPT-4o, ChatGPT-5 Thinking, Gemini 2.5 Pro and DeepSeek-V3.2, were evaluated by four board-certified oncologists against the actual MDT treatment decisions. Additionally, the LLM-generated recommendations were also rated across five pre-specified domains: clarity, clinical applicability, coverage, explanation and support with evidence, and guideline concordance.

**Results:**

In this study, inter-rater reliability was acceptable to good, supporting the consistency of expert evaluation. ChatGPT-5 Thinking showed the strongest and most consistent overall performance, followed by ChatGPT-4o, while Gemini 2.5 Pro and DeepSeek-V3.2 were rated less favorably. Differences between LLMs were statistically significant across all evaluated domains. Performance differences appeared most clinically relevant in more complex scenarios, particularly when consideration of adjuvant or systemic treatment strategies was required.

**Conclusion:**

The findings of this study suggest that selected LLMs may have a supportive role in everyday melanoma MDT practice particularly in oncology centers with limited resources. However, the current results do not support the use of LLM-generated recommendations as independent treatment decisions, and further prospective studies are required before LLM-assisted treatment recommendations can be safely integrated into the MDT workflow.

## 1 Introduction

Malignant melanoma, the most lethal form of skin cancer, and an important global oncologic challenge, continues to show a rising incidence worldwide. In 2020, an estimated 325 000 new melanoma cases and 57 000 deaths were recorded worldwide, and if 2020 rates continue, the burden from melanoma is estimated to increase to 510 000 new cases (a roughly 50% increase) and to 96 000 deaths (a 68% increase) by 2040 (1). The increasing incidence of melanoma has not been followed by a rise in mortality, likely reflecting improvements in public awareness of skin self-examination and early recognition of suspicious cutaneous lesions, which facilitates diagnosis at an early stage, when surgical resection is associated with a 98% 5-year survival rate. On the other hand, within stage III, 5-year survival rates range from 20% to 70%, depending primarily on the nodal tumor burden (2). Historically, distant metastatic melanoma is marked with poor prognosis, but emerging effective systemic therapies present hope for changing the survival of patients with stage IV melanoma.

The task of clinicians involved in melanoma management places strong emphasis on prevention and early detection, followed by multidisciplinary decision-making requiring close collaboration among oncologists, dermatologists, surgeons, pathologists and radiologists, in accordance with relevant guidelines for the diagnosis, treatment and follow-up of patients with malignant melanoma. In this context, multidisciplinary tumor board (MDT) has become the clinical setting that allows translating rapidly changing evidence into individualized recommendations for each melanoma patient (3, 4). In routine clinical practice, this represents a complex process dependent on a high level of clinician commitment as well as substantial resources, which can become particularly challenging when any of these elements are lacking. These constraints are often encountered in medical centers in low- and middle-income countries (LMICs), where melanoma care is further complicated by workforce shortages, a high patient burden per oncologist, and limited access to immunotherapy and targeted therapy for all eligible patients and disease stages (5).

Against this backdrop, artificial intelligence (AI), particularly large language models (LLMs), have emerged as potentially valuable support tools in oncology practice including clinical decision support (6–8). LLMs have been evaluated in experimental settings for clinical cases showing promising results in suggesting potential diagnoses, pointing users to the correct guidelines or directly proposing treatments, but still with many challenges including biases, keeping systems up to date with clinical evidence, interoperability, and continuous improvements (9). This is of special relevance in resource-constrained environments, where LLMs may serve as valuable supportive tools for MDTs by enhancing the capacity to deliver high-quality individualized care (10, 11).

This study therefore evaluated four different LLMs for melanoma treatment recommendations in a real-world MDT cohort in a LMIC-setting by comparing real-world MDT treatment recommendations for newly diagnosed malignant melanoma patients with recommendations generated by LLMs. Through comparison with actual MDT recommendations and independent oncologist ratings, this study also examined the clinical relevance, guideline concordance, and practical applicability of contemporary LLMs in modern melanoma care.

## 2 Materials and methods

### 2.1 Patient cohort

This retrospective, single-center cohort study included 151 consecutive patients with newly diagnosed malignant melanoma who underwent initial treatment discussion at the multidisciplinary tumor board (MDT) specialized in melanoma care at the Clinic for Oncology and Radiotherapy, University Clinical Center Tuzla, Bosnia and Herzegovina, from 1 January 2020 to 31 December 2024. Inclusion criteria comprised treatment-naïve patients with histologically confirmed cutaneous melanoma who were presented at the MDT to define the initial treatment strategy. Exclusion criteria were age <18 years, recurrent melanoma, multiple primary malignancies, participation in clinical trials, or insufficient/incomplete clinical data required for the study analysis.

Patient records and MDT documents were used to extract demographic, clinical and pathological variables, including year of diagnosis, sex, age, comorbidities, ECOG performance status, primary tumor anatomical site, detailed histopathology findings (diagnosis, Breslow thickness, ulceration, Clark level, mitotic rate, microsatellites, satellite and in-transit metastases, neurotropism, lympho-vascular invasion, histological subtype) as well as data on sentinel lymph node biopsy (SLNB), *BRAF* V600E mutation status, AJCC stage at presentation, presence of visceral metastases, initial diagnostic/treatment procedures (e.g. diagnostic biopsy, excision). For each patient, the MDT’s final consensus management recommendation was extracted from the official MDT record. Potential modalities included surgery, systemic therapy, radiotherapy, or multimodal approaches. Baseline characteristics of the patient cohort are depicted in **Table 1**.

**Table 1:** Baseline characteristics.

Variable	N (%)
Sex	
Female	62 (41.1)
Male	89 (58.9)
ECOG performance status	
0–1	140 (92.8)
2	4 (2.6)
3	7 (4.6)
AJCC stage at presentation	
0	9 (6.0)
IA	10 (6.6)
IB	20 (13.2)
IIA	14 (9.3)
IIB	27 (17.9)
IIC	16 (10.6)
IIIA	0
IIIB	1 (0.7)
IIIC	25 (16.5)
IIID	2 (1.3)
IV	27 (17.9)
Primary tumor site	
Head/Neck	33 (21.8)
Trunk	62 (41.1)
Upper extremity	19 (12.6)
Lower extremity	25 (16.5)
Other/unspecified	12 (8.0)
Histologic subtype	
Nodular melanoma	60 (39.8)
Superficial spreading melanoma	15 (9.9)
Lentigo maligna melanoma	2 (1.3)
Desmoplastic melanoma	1 (0.7)
Spitzoid melanoma	2 (1.3)
Not otherwise specified	71 (47.0)
Breslow thickness category	
0	8 (5.3)
<0.8 mm	4 (2.6)
0.8–1.0 mm	10 (6.6)
1.0–2.0 mm	23 (15.3)
2.0–4.0 mm	37 (24.5)
>4.0 mm	61 (40.4)
Unspecified	8 (5.3)
Ulceration	
Present	85 (56.3)
Absent	56 (37.1)
Not applicable	10 (6.6)
*BRAF* V600E status	
Mutated	40 (26.5)
Wild type	46 (30.5)
Unknown	65 (43.0)
SLNB	
Positive	34 (22.5)
Negative	80 (53.0)
Not done	37 (24.5)
Number of positive lymph nodes	
0	81 (53.7)
1	18 (11.9)
2	7 (4.6)
≥3	9 (5.9)
Unknown/Not done	36 (23.9)
MDT treatment recommendation	
Surgery alone	102 (67.6)
Surgery + adjuvant therapy	5 (3.3)
Immunotherapy	20 (13.2)
Immunotherapy + radiotherapy	4 (2.6)
BRAF/MEK	7 (4.6)
BRAF/MEK + radiotherapy	6 (4.0)
Supportive care	6 (4.0)
Radiotherapy + supportive care	1 (0.7)
Number of MDT treatment options	
1	69 (45.7)
2	42 (27.8)
≥3	40 (26.5)

To protect patient confidentiality, all patient records were anonymized prior to analysis, and direct identifiers were removed. No patient-identifiable information was entered into the LLM. Informed consent was obtained from all patients upon admission, in accordance with the standard institutional procedure whereby patients sign a general consent form authorizing the use of their medical data for research purposes. This study was approved by the Ethics Committee of the University Clinical Center Tuzla (Approval No. 02-09/2-133-3/25).

### 2.2 Study design

Four large language models (LLMs) were evaluated: ChatGPT-4o (OpenAI), ChatGPT-5 Thinking (OpenAI), Gemini 2.5 Pro (Google DeepMind (Google)), and DeepSeek-V3.2 (DeepSeek) using their publicly available interfaces under standard settings. ChatGPT-4o outputs were generated between 30 August and 1 September 2025, Gemini 2.5 Pro between 30 September and 5 October 2025, DeepSeek-V3.2 and ChatGPT-5 Thinking outputs between 5 and 7 October 2025. ChatGPT-4o and ChatGPT-5 Thinking were accessed through the ChatGPT web interface, Gemini 2.5 Pro through the Gemini web interface, and DeepSeek-V3.2 through the DeepSeek web interface. No API access, model fine-tuning, or custom generation parameters were used. The evaluation was designed to reflect a pragmatic, clinician-facing use scenario rather than a controlled software-engineered LLM pipeline.

National Comprehensive Cancer Network (NCCN) and European Society for Medical Oncology (ESMO) melanoma guidelines corresponding to the year of each patient’s diagnosis were uploaded to the LLMs as PDF documents within the chat interface. This approach should be distinguished from formal retrieval-augmented generation (RAG), because no independent document preprocessing, chunking, indexing, vector database, retriever selection, retrieval logging, or source-passage validation was implemented. Prior to case-level prompting, all LLMs were instructed to generate clear, multidisciplinary tumor board–style recommendations based on these documents and aligned with NCCN/ESMO guidance for the patient’s year of diagnosis. All text data were translated into English. The same standardized prompts were used for all models and each case was evaluated in a different chat window. An example of prompt: “Act as a multidisciplinary oncology tumor board. Provide melanoma treatment recommendations for newly diagnosed patient based on NCCN/ESMO guidelines (year of diagnosis 2020.). Patient case: age 76, female, treatment done: excision, re-excision, lymph node extirpation. Histopathology report: Melanoma malignum exulceratum cutis, pT3b. Histopathology report after re-excision and lymph node extirpation without malignant cells. Location: trunk. *BRAF* negative. SLNB negative. Ulceration: present. Clark level: IV. Mitotic rate: 5 mitoses/10HPF. Microsatellites: absent. In-transit metastases: absent. Satellite metastases: absent. Neurotropism: absent. Lympho-vascular invasion: absent. Histological subtype: nodular. Comorbidities: arterial hypertension. ECOG: 0.

Four board-certified medical oncologists from three referral centers independently evaluated the treatment plans proposed by the MDT and those generated by the LLMs. All raters had more than 10 years of clinical experience and were routinely involved in the management of melanoma patients and melanoma-related MDT decision-making. In order to reduce the possible institutional biases, three out of four oncologists were based at external institutions and had no formal affiliation with the study center.

All LLM-generated recommendations were evaluated against the MDT treatment decisions. For each case, oncologists independently rated the extent of agreement between LLM-generated recommendation and the MDT decision using a 4-point Likert scale (1: Strongly disagree; 2: Disagree; 3: Agree; 4: Strongly agree). A 4-point Likert scale was selected as a forced-choice format, rather than a scale including a neutral midpoint, to elicit directional judgments regarding both overall concordance and domain-specific quality of the LLM-generated recommendations, while reducing reliance on neutral responses. In addition, the characteristics of the LLM-generated recommendations were also rated using the same 4-point Likert scale across five pre-specified domains:

1. Clarity and coherence (“Is the answer logically structured, clinically appropriate in tone, and understandable for MDT use?”),
2. Clinical applicability (“Could the recommendation be directly applied in practice? Were there ambiguities that might cause clinical uncertainty?”),
3. Coverage (“Were all aspects of management considered (surgery, radiotherapy, systemic therapy, palliative care, follow-up)?”),
4. Explanation and support with evidence (“Did the output cite guidelines, clinical trials, or provide explicit justification?”),
5. Guideline concordance (“Are the recommendations consistent with NCCN/ESMO guidelines for the specified year? Are staging and treatment pathways correct?”).

The oncologist raters were blinded to the LLM and its version throughout grading.

### 2.3 Statistics

Ratings were retained at the individual oncologist rater level. Total scores were calculated to reflect agreement levels. Inter-rater reliability was assessed using Gwet’s AC2, Krippendorff’s alpha, and Cronbach’s alpha. Differences between the four LLMs across domains were evaluated using Friedman tests with Dunn’s *post-hoc* pairwise comparisons and Bonferroni correction. All statistical analyses were performed using R software (version 4.5.1; R Foundation for Statistical Computing, Austria). A p value < 0.05 was considered statistically significant.

## 3 Results

### 3.1 Patient characteristics

A total of 151 melanoma cases were included in the analysis. Males represented 58.9% of the population. Preserved functional status (ECOG 0-1) was documented in 92.8% of the patients. All disease stages from 0 to IV were represented with stage IIB (17.9%), stage IIIC (16.5%), and stage IV (17.9%) as the largest subgroups. The trunk was the most frequent primary tumor site, observed in 41.1% of cases. *BRAF* V600E status was unknown in 42.3% of patients, while 26.5% had *BRAF*-mutant disease and 30.5% were *BRAF* wild-type. The predominant MDT recommendation was surgical treatment alone, accounting for 67.6% of cases, followed by immunotherapy in 13.2% of cases. The characteristics of the patient cohort are given in **Table 1**. (Detailed baseline clinicopathologic characteristics of the study cohort are depicted in the **Supplementary Table 1** and **Supplementary Figure 1**).

### 3.2 Expert ratings and inter-rater reliability

Four raters assessed LLM-generated recommendations for 151 patients, yielding 604 individual scores per LLM and additionally for each of five pre-specified domains. Reliability of the ratings was assessed using multiple statistical measures, enabling a comprehensive evaluation of the consistency and agreement among the raters. The results show that there was high agreement among the raters, confirmed by Gwet AC2 0.704 (95% CI: 0.668–0.741, parametric normal approximation), while Krippendorff’s α 0.633 (95% CI: 0.604–0.669, bootstrap method) indicated acceptable internal consistency. Cronbach’s alpha further confirmed satisfactory internal consistency of the scale 0.76 (95% CI: 0.70–0.81, Feldt method). Summary of oncologist ratings is depicted in **Table 2**.

**Table 2:** Summary of oncologist ratings.

Model	Mean score	SD	Count ≤2	% ≤2	Count >2	% >2
ChatGPT-4o	3.258278	0.6810739	34	5.6	570	94.4
ChatGPT-5 Thinking	3.298013	0.6469185	39	6.5	565	93.5
Gemini 2.5 Pro	3.134106	0.7240679	115	19.0	489	81.0
DeepSeek-V3.2	3.039735	0.8155275	125	20.7	479	79.3

### 3.3 Overall comparative performance of LLMs

**Figure 1:**
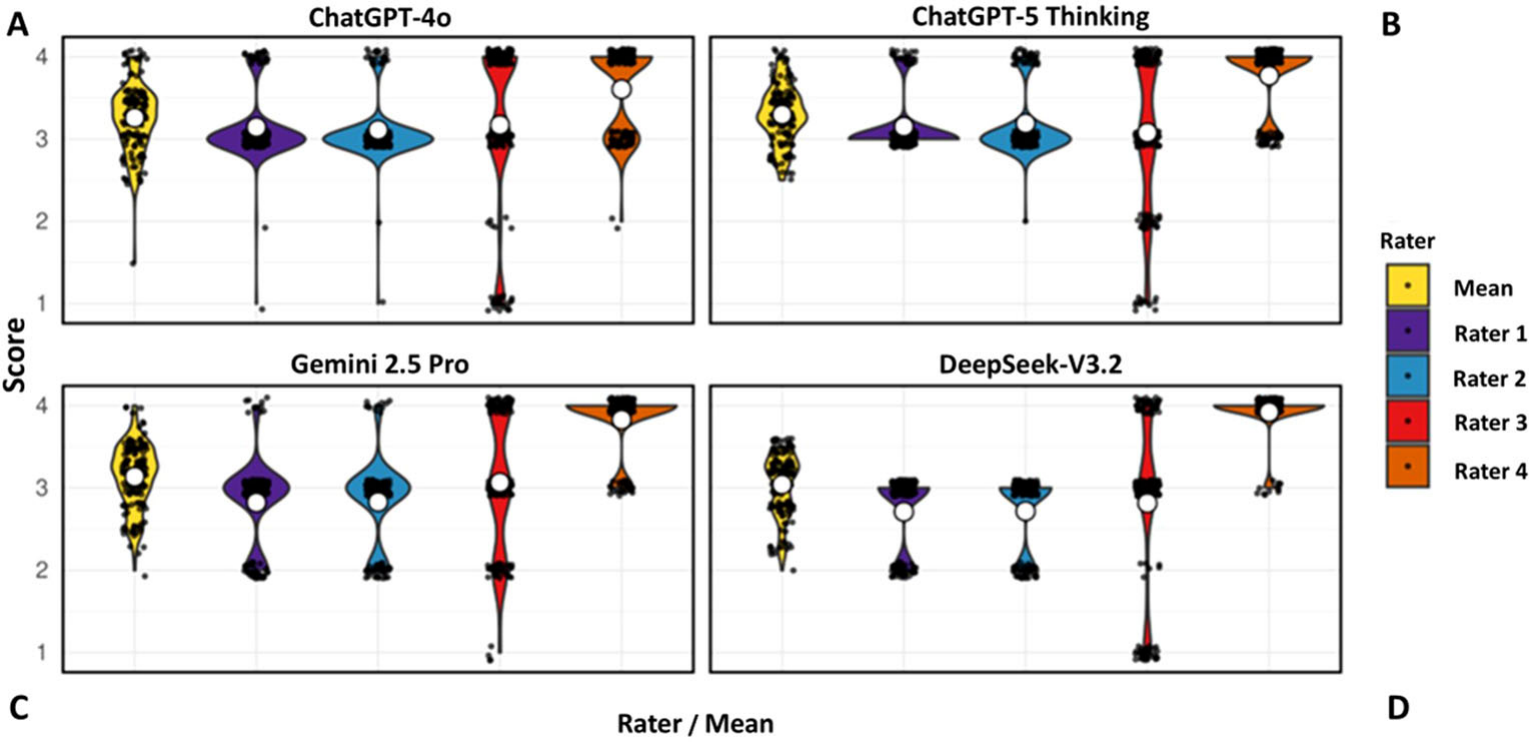
Distribution of expert agreement scores across the four evaluated LLMs. **(A)** ChatGPT-4o; **(B)** ChatGPT-5 Thinking; **(C)** Gemini 2.5 Pro; **(D)** DeepSeek-V3.2. Violin plots display the distribution of agreement scores for each of the four expert raters and their average. Scores ranged from 1 (strongly disagree) to 4 (strongly agree). White dots indicate the mean score for each rater. .

ChatGPT-5 Thinking achieved the highest mean score (3.298, SD 0.647), followed by ChatGPT-4o (3.258, SD 0.681), Gemini 2.5 Pro (3.134, SD 0.724) and DeepSeek-V3.2 (3.040, SD 0.816). Ratings ≤2 were not frequent for ChatGPT-5 Thinking and ChatGPT-4o (6.5% and 5.6%), but more common for Gemini 2.5 Pro and DeepSeek-V3.2 (19.0% and 20.7%). Violin plot of rater scores depicted in **Figure 1**.

### 3.4 Comparative performance across evaluation domains

Statistical analysis identified ChatGPT-5 Thinking as the best-performing LLM across all five evaluation domains. The Friedman test showed highly significant differences among the four LLM versions in every category (p < 0.001). Regarding the domain “Clarity and Coherence”, ChatGPT-5 Thinking performed best with a median score of 4 and was statistically superior to other LLMs: ChatGPT-4o, Gemini 2.5 Pro and DeepSeek-V3.2 (p < 0.001). For domain “Clinical Applicability”, both ChatGPT-4o and ChatGPT-5 Thinking had a median score of 4 and no significant difference was observed between those two LLM versions (p = 0,611). ChatGPT-5 Thinking also performed best in domain “Coverage” with a median score of 4 and a high minimal score of 3, demonstrating significant superiority over other LLM (p<0,001). Although all used LLMs had the same median score of 3 in domain “Explanation and Support with Evidence”, *post hoc* testing showed that the rank distribution for ChatGPT-5 Thinking remained significantly better in comparison with, Gemini 2.5 Pro and DeepSeek-V3.2 (p < 0,001). Finally, ChatGPT-5 Thinking had the best results in domain “Guideline Concordance”, with a median score of 4, showing a statistically significant advantage over all other LLMs, indicating that ChatGPT-5 Thinking was the most reliable LLM among those four used, in terms of adherence to clinical standards (**Table 3**, **4**). *Post-hoc* pairwise comparisons between LLMs across evaluation domains using Dunn’s test with Bonferroni correction are depicted in the **Supplementary Table 2**.

**Table 3:** Distribution of expert ratings for each LLM version across evaluation domains.

Domain	LLM	Median (IQR)	Min	Max
Clarity and coherence	ChatGPT-4o	3 (1)	2	4
	ChatGPT-5 Thinking	4 (1)	2	4
	Gemini 2.5 Pro	3 (1)	2	4
	DeepSeek-V3.2	3 (1)	1	4
Clinical applicability	ChatGPT-4o	4(1)	1	4
	ChatGPT-5 Thinking	4(1)	1	4
	Gemini 2.5 Pro	3(1)	1	4
	DeepSeek-V3.2	3(1)	1	4
Coverage	ChatGPT-4o	3(1)	1	4
	ChatGPT-5 Thinking	4(1)	3	4
	Gemini 2.5 Pro	3(1)	2	4
	DeepSeek-V3.2	3(1)	2	4
Explanation and support with evidence	ChatGPT-4o	3(1)	2	4
	ChatGPT-5 Thinking	3(1)	2	4
	Gemini 2.5 Pro	3(1)	2	4
	DeepSeek-V3.2	3(1)	2	4
Guideline concordance	ChatGPT-4o	3(1)	1	4
	ChatGPT-5 Thinking	4(1)	2	4
	Gemini 2.5 Pro	3(1)	2	4
	DeepSeek-V3.2	3(1)	2	4

**Table 4:** Results of the Friedman test comparing LLM versions across evaluation domains based on expert rater assessments.

Domain	Friedman χ²	df	p-value
Clarity and coherence	103.291	3	p < 0.001
Clinical applicability	135.636	3	p < 0.001
Coverage	114.287	3	p < 0.001
Explanation and support with evidence	186.433	3	p < 0.001
Guideline concordance	62.331	3	p < 0.001

### 3.5 Performance across clinicopathologic subgroups

Descriptive subgroup analyses showed that ChatGPT-5 Thinking had generally the most favorable ratings across AJCC stage groups and TNM categories, followed by ChatGPT-4o, while other two LLMs, Gemini 2.5 Pro and DeepSeek-V3.2, were less favorably evaluated. Similar pattern was also observed across other characteristics including *BRAF* mutation status, ulceration, Breslow thickness, sentinel lymph node biopsy status, number of positive lymph nodes, primary tumor site, histologic subtype, as well as ECOG performance status, comorbidity burden, MDT recommendation category, and complexity of treatment options presented by number of possible treatment options. Differences between LLMs appeared more clinically meaningful in subgroups associated with greater treatment complexity, particularly when consideration of adjuvant or systemic treatment strategies was required. These findings should be interpreted as descriptive and hypothesis-generating rather than confirmatory given the limited number of patients in several subgroups. Performance analysis across clinicopathologic subgroups is depicted in the **Supplementary Table 3**.

## 4 Discussion

In this real-world melanoma MDT cohort, LLM-generated treatment recommendations demonstrated generally favorable expert-rated overall agreement with MDT-decisions, while also revealing clinically meaningful differences in performance across LLM versions. Inter-rater reliability was acceptable to good, with Gwet’s AC2 of 0.704, Krippendorff’s α of 0.633, and Cronbach’s alpha of 0.76, suggesting that the observed differences between models were unlikely to be explained primarily by inconsistency among raters.

ChatGPT-5 Thinking achieved the highest overall mean score and the most favorable overall performance profile, followed by ChatGPT-4o, particularly in clinical applicability, and with a low proportion of poorly concordant ratings. In contrast, Gemini 2.5 Pro and DeepSeek-V3.2 were associated with a substantially higher proportion of low-agreement ratings. Taken together, these findings suggest that the potential utility of large language models as supportive tools in melanoma multidisciplinary tumor board practice depends on the used model, and that these models should not be regarded as interchangeable. In this study, consistency refers not only to agreement among raters, but also to the relative stability of model ranking across different clinicopathologic subgroups.

It should also be emphasized that the endpoint of this study was not objective treatment accuracy nor patient outcomes, but rather expert-rated concordance between LLM-generated recommendations and actual MDT-decisions, together with assessment of clarity, clinical applicability, comprehensiveness, explanation, and guideline concordance. In other words, although a model may generate a recommendation that appears clinically reasonable and is rated favorably by experts, this in itself is not evidence of improved patient outcomes or of a safe replacement for multidisciplinary decision-making. Rather, the present findings indicate that some LLMs are better than others in generating the form of recommendations that melanoma experts would consider acceptable in routine practice.

Given that the predominant MDT-decision in this cohort was surgical treatment, and that nearly half of the patients had only one realistic treatment option, concordance in such cases was likely easier to achieve because of the limited therapeutic complexity. In contrast, descriptive analyses across clinicopathologic subgroups suggest that differences between LLMs became more pronounced in clinically more complex scenarios, particularly when treatment decisions involved adjuvant therapy, nodal metastases, systemic treatment, *BRAF* status, or multiple acceptable management options.

In this context, the findings support a cautious interpretation of concordance between LLMs and MDT-decisions. If such models were to be used in real-world clinical practice, small differences in mean scores between models might be less important than the frequency of clearly unsatisfactory responses. Accordingly, the lower proportion of poorly concordant ratings observed among the higher-ranked models is clinically relevant, even though all evaluated systems were capable of generating at least some acceptable recommendations. At the same time, because lower-rated recommendations were observed across all models, none of the evaluated LLMs can be considered sufficiently reliable for unsupervised use in clinical practice without expert oversight. Importantly, no LLM-generated recommendation was identified as clearly hallucinatory or factually incorrect with respect to the input data. Lower expert ratings instead reflected incomplete or insufficiently detailed responses, particularly in staging accuracy and treatment pathway selection for DeepSeek-V3.2 and Gemini 2.5 Pro. This distinction is clinically relevant: the predominant failure mode in this cohort was inadequacy rather than error, suggesting that in a supervised MDT setting, the primary risk of lower-performing models may be the omission of clinically important considerations rather than the generation of clearly harmful recommendations.

These findings should also be interpreted in the context of the broader literature on AI in skin malignancies, which is considerably more developed in diagnostics, image analysis, and other supportive clinical applications than in real-world treatment planning support (12). Regarding this, literature specifically addressing melanoma treatment and the role of LLMs in MDT-decision support remains limited. Earlier studies suggest that LLMs can generate plausible treatment recommendations, but their depth, specificity, and reliability remain variable. In their systematic review, Zarfati et al. identified a total of nine studies on the use of LLMs in melanoma, only one of which specifically addressed treatment advice (13). That being mentioned, Mu et al. reported that ChatGPT slightly outperformed BARD and BingAI in providing more reliable and evidence-based clinical recommendations, although important limitations in depth and specificity remained (14). In this context, the present study adds to the existing literature by evaluating several contemporary LLMs against real melanoma MDT-decisions, rather than isolated simulated cases or general educational outputs, while assessing not only overall concordance, but also clarity, clinical applicability, comprehensiveness, explanation, and guideline concordance.

Evidence from other oncologic studies beyond melanoma also supports a measured interpretation of these findings. Studies in other malignancies suggest that concordance LLM-generated recommendations and MDT-decisions tends to be higher in more structured, protocol-driven, and guideline-defined cases, and lower in more heterogeneous and clinically complex scenarios. In a colorectal cancer MDT-study, Chatziisaak D. et al. reported substantial concordance between ChatGPT-4 recommendations and tumor board decisions (15). Similarly, Birsin et al. evaluated ChatGPT-5 against MDT recommendations in 179 stage II colon cancer patients and found moderate overall agreement (70.4%, κ = 0.542), which improved to substantial in binary adjuvant versus observation decisions (91.1%, κ = 0.719); notably, agreement decreased in elderly and frail patients with multiple risk factors (16), a pattern consistent with our observation of weaker LLM performance in more complex clinicopathologic subgroups. Likewise, in thoracic malignancies, Zabaleta et al. reported 76% concordance and a kappa value of 0.59, supporting the potential value of LLM-assisted decision support in well-defined clinical settings (17). By contrast, Li et al. reported more limited agreement in a sarcoma ring-trial setting, in which LLM-generated recommendations matched the most frequent MDT-recommendation in only 20%–60% of cases, underscoring the challenges of decision-making in more clinically complex situations (18). In a related sarcoma study, Dehdab et al. evaluated ChatGPT-4o across 152 soft tissue sarcoma cases using a five-domain scoring framework, comparable in structure to our study, and found that while clinical contextualization was the strongest domain, performance in treatment sequencing and chemotherapy selection was substantially lower (19), suggesting that LLMs may be better at framing clinical context than at providing precise technical guidance. Karabuğa et al. likewise found low agreement between ChatGPT-4o and tumor board decisions despite good inter-rater reliability, further highlighting the limitations of current models in heterogeneous real-world oncology practice (20). The importance of evaluating LLMs beyond simple concordance is underscored by Yang et al., who linked LLM-MDT agreement to actual survival outcomes in over 13,000 HCC patients. Notably, concordance rates between LLM recommendations and physician decisions were low across all models (31.1% for ChatGPT-4o, 32.7% for Gemini 2.0, and 26.8% for Claude 3.5), yet that concordance was associated with improved survival in early-stage but not advanced-stage disease, with physicians outperforming LLMs in complex cases by incorporating liver function parameters that guideline-based models deprioritized (21). Taken together, these studies indicate that the clinical value of LLM-supported MDT-decision making depends not only on the model itself, but also on the type of malignancy, the degree of case heterogeneity, and the extent to which clinical decision-making is standardized rather than highly individualized. The present findings therefore align with studies showing better concordance between LLM and MDT in more structured clinical contexts, but do not support the assumption that such concordance is uniformly high in oncology.

This is particularly relevant in melanoma treatment, where contemporary management based on NCCN and ESMO guidelines increasingly requires nuanced consideration of disease stage, adjuvant therapy, nodal burden, molecular profile, and neoadjuvant treatment (3, 4). Such cases are less algorithmic than those in which management is primarily surgery-based and are therefore more demanding for LLM reasoning.

One of the important findings of this study is that the domain of explanation and support with evidence remained only moderate across all LLMs. This is particularly important because a recommendation may appear clinically valid even when the reasoning on which it is based is incomplete or insufficiently explicit. In real-world clinical practice, treatment recommendations are more useful when their logic is explicit, easily verifiable, and directly comparable with guidelines and relevant clinical trial evidence. The multidimensional evaluation framework used in this study can therefore be regarded as a strength, as it shows that apparent concordance with MDT-decisions does not necessarily imply high-quality explanation. This observation is also consistent with the broader oncology literature. Hao et al., in a 2025 systematic review and meta-analysis, identified 56 studies across 15 cancer types and concluded that most evaluations primarily emphasized accuracy and appropriateness, whereas safety, potential harm, and clarity were assessed less consistently (22).

A similar pattern of better concordance in more standardized cases and weaker concordance in more clinically individualized scenarios was also observed in a previous study from the same center in breast cancer. Although direct comparison is limited by differences in malignancy type, treatment modalities, and the evaluated LLMs, those two studies together suggest that assessing concordance between LLM-recommendations and MDT-decisions is relevant even in real-world resource-constrained settings (23).

Several limitations of the present study should be acknowledged. First, this was a retrospective single-center study, and neither patient outcomes nor prospectively measured changes in MDT efficiency were assessed, which should be considered in the context of rapidly evolving LLM versions. Importantly, publicly available LLMs are continuously updated by their vendors. Therefore, the present findings reflect the model versions accessible at the time of evaluation and may not fully generalize to later iterations of the same systems. Because the study was conducted at a single center, broader applicability of these findings beyond the study setting may be limited. Moreover, LLMs generated recommendations using standardized prompt and uploaded guideline documents, meaning that the observed performance differences between LLMs may partly reflect differences in how effectively models responded to the specific prompt and uploaded materials, rather than intrinsic model capability alone. The used prompting strategy combined zero-shot prompting, role prompting, and open-ended output generation. No examples of correct recommendations were provided, the models were instructed to act as a multidisciplinary oncology tumor board, and no rigid schema-enforced template was imposed. This approach was chosen to mirror real-world clinical practice, in which decisions are being made based on concrete documentation and with varying levels of available context. However, prompt formulation itself can affect output quality, completeness, interpretability, and reproducibility. Open-ended prompting may allow clinically flexible reasoning, but on the other hand may also increase variability in structure, coverage, and explicit justification. Schema-enforced prompting could improve comparability across models and facilitate structured scoring, but might constrain clinical nuance. Zero-shot prompting used in this study reflects realistic clinician-facing use and avoids example-driven bias but may increase output variability and also does not guide models toward an ideal answer format. Role prompting encourages clinically structured, MDT-style responses. Although, role prompting may improve style and framing, it does not guarantee factual accuracy or guideline concordance. Prompting methods such as chain-of-thought prompting, nor prompt repetition were not used in this study; therefore, the study could not assess whether these methods would improve guideline concordance or response stability (24–26). Also, the used approach in this study with uploading guideline documents into a chat interface should not be interpreted as a controlled retrieval-augmented generation (RAG). Unlike explicit RAG pipelines, retrieval behavior, document coverage, or token-level grounding could not be verified, and therefore it cannot be claimed to which extent LLM-recommendations were derived directly from the uploaded guidelines (27, 28).

However, our approach mirrors real-world clinical practice, in which decisions are being made based on concrete documentation and with varying levels of available context, and may therefore also be viewed as a practical strength. It should also be noted that while aggregate inter-rater reliability was acceptable to good, case-level analysis of the raw data revealed that notable inter-rater discordance was infrequent and not randomly distributed. Disagreements were primarily observed in cases where LLM recommendations included systemic treatment options, particularly immunotherapy, that are not uniformly available across all participating institutions, with raters from centers with more restricted therapeutic access tending to rate clinical applicability of such recommendations lower. This pattern reflects a genuine and clinically meaningful source of variability: the practical applicability of a treatment recommendation is inherently context-dependent, and what constitutes an appropriate recommendation in one setting may not be directly transferable to another. Raters were kept blinded and did not discuss individual cases, as *post-hoc* consensus would have risked obscuring this real-world heterogeneity. Aggregate reliability metrics should therefore be interpreted with the understanding that some of the observed variance between raters reflects institutional differences in treatment availability rather than rater inconsistency per se.

Regarding further limitations, subgroup analyses were descriptive and should be interpreted cautiously, as the study was neither designed nor statistically powered to support definitive subgroup-level conclusions. The inclusion of only newly diagnosed, treatment-naïve patients with primary cutaneous melanoma provided a standardized initial MDT decision point, but may also have favored higher LLM-MDT concordance, particularly because surgery alone was the predominant MDT recommendation. Therefore, the findings may not generalize to recurrent and previously treated melanoma scenarios. Future studies should specifically evaluate LLM performance in these patient groups and treatment settings. Regarding the scale used, the absence of a neutral midpoint of used 4-point Likert scale may have influenced rating distributions by requiring raters to lean toward agreement or disagreement, which should be considered when interpreting the results (29). Finally, concordance with MDT-decisions should not be interpreted as evidence of the clinical superiority, safety, or patient outcome benefit of any model, but rather as an expert-rated indicator of clinical applicability and alignment with real-world MDT decision-making.

Overall, the findings of this study support a cautious and supervised role of LLMs in melanoma MDT practice, suggesting that selected models may be useful for structuring clinical information and presenting treatment options aligned with current guidelines. However, these findings do not support autonomous treatment decision-making. Within the scope of this study, it can be consequently concluded that LLMs may have a role in supporting clinical decision-making in routine practice, but final treatment decisions should therefore remain the responsibility of the MDT.

## 5 Conclusions

In this study based on a real-world melanoma MDT cohort, expert ratings of LLM-generated melanoma treatment recommendations demonstrated substantial reliability and identified significant performance differences across four LLMs regarding clinical applicability and guideline concordance, highlighting the potential of a supportive role of LLMs in everyday MDT practice. Additionally, the study results suggest potential utility in oncology centers with limited resources. Although ChatGPT-5 Thinking showed the strongest and most consistent overall performance, the occurrence of lower ratings in other LLMs, as well as the absence of clinical outcome validation, indicates that LLM-generated recommendations should not be used as independent treatment decisions. Further prospective studies across additional LLMs using locked model versions, with clear oversight of AI-assisted medical documentation and structured safety monitoring, are needed before safely implementing the LLMs into the improved MDT workflow.

## Data Availability

The raw data supporting the conclusions of this article will be made available by the authors, without undue reservation.
