# Supplementary material for: Clinical evaluation of large language model recommendations in melanoma: comparison with multidisciplinary tumor board decisions in a real-world cohort
Source: Front Oncol. 2026 Jun 29;16:1856390. doi: 10.3389/fonc.2026.1856390 (PMC13357115; doi:10.3389/fonc.2026.1856390)
Supplement: Supplementary file 1 [file DataSheet1.docx]

**Clinical Evaluation of Large Language Model Recommendations in Melanoma: Comparison with Multidisciplinary Tumor Board Decisions in a Real-World Cohort**

**Supplementary Material**

**Supplementary Figure 1. Age distribution**


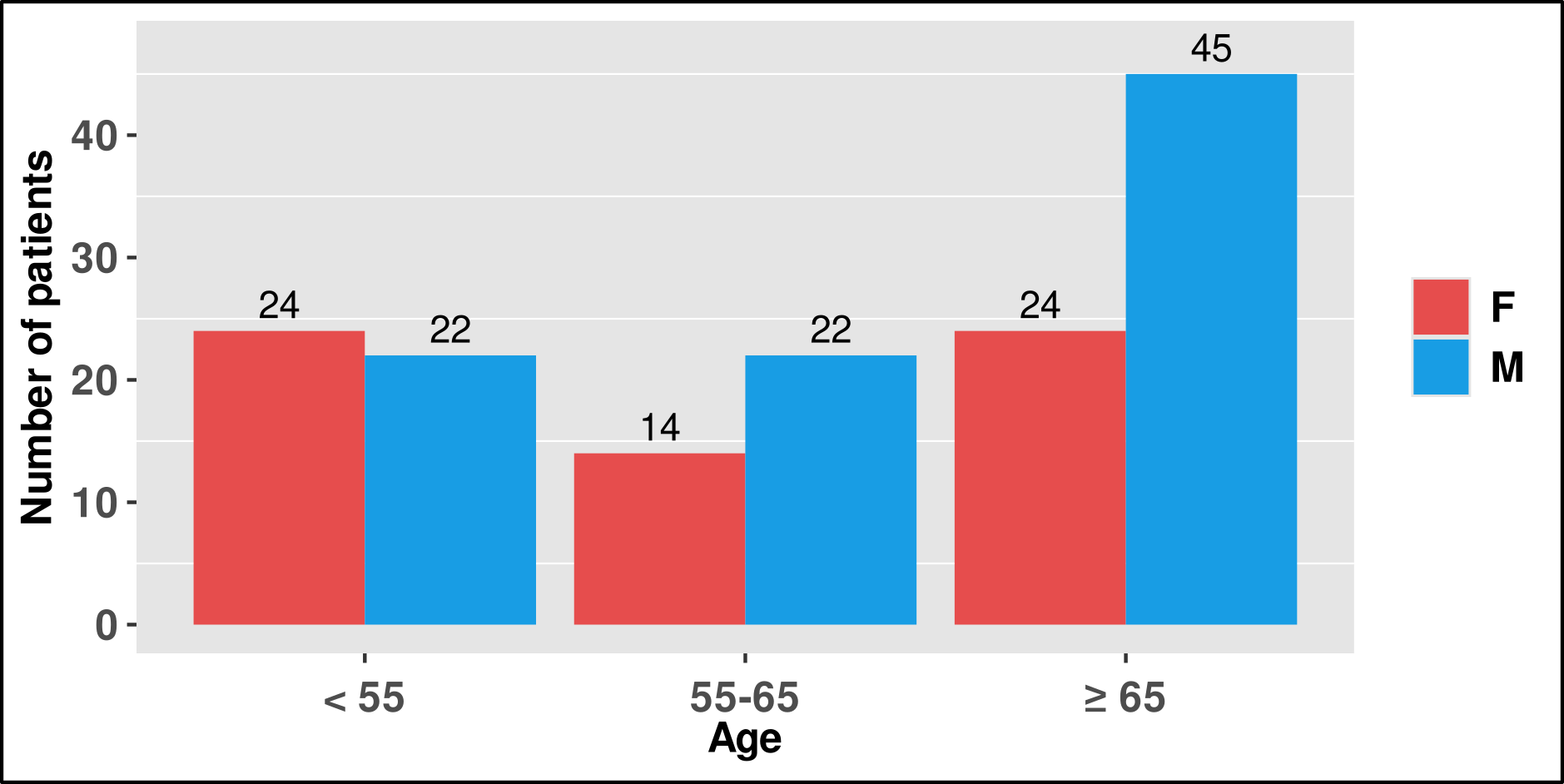


**Supplementary Table 1. Baseline characteristics**

| **Variable** | **N (%)** |
| --- | --- |
| **Sex** | |
| Female | 62 (41.1) |
| Male | 89 (58.9) |
| **ECOG performance status** | |
| 0–1 | 140 (92.8) |
| 2 | 4 (2.6) |
| 3 | 7 (4.6) |
| **AJCC stage at presentation** | |
| 0 | 9 (6.0) |
| IA | 10 (6.6) |
| IB | 20 (13.2) |
| IIA | 14 (9.3) |
| IIB | 27 (17.9) |
| IIC | 16 (10.6) |
| IIIA | 0 |
| IIIB | 1 (0.7) |
| IIIC | 25 (16.5) |
| IIID | 2 (1.3) |
| IV | 27 (17.9) |
| **Primary tumor site** |  |
| Head/Neck | 33 (21.8) |
| Trunk | 62 (41.1) |
| Upper extremity | 19 (12.6) |
| Lower extremity | 25 (16.5) |
| Other/unspecified | 12 (8.0) |
| **Histologic subtype** | |
| Nodular melanoma | 60 (39.8) |
| Superficial spreading melanoma | 15 (9.9) |
| Lentigo maligna melanoma | 2 (1.3) |
| Desmoplastic melanoma | 1 (0.7) |
| Spitzoid melanoma | 2 (1.3) |
| Not otherwise specified | 71 (47.0) |
| **Breslow thickness category** | |
| 0 | 8 (5.3) |
| <0.8 mm | 4 (2.6) |
| 0.8–1.0 mm | 10 (6.6) |
| 1.0–2.0 mm | 23 (15.3) |
| 2.0–4.0 mm | 37 (24.5) |
| >4.0 mm | 61 (40.4) |
| Unspecified | 8 (5.3) |
| **Ulceration** | |
| Present | 85 (56.3) |
| Absent | 56 (37.1) |
| Not applicable | 10 (6.6) |
| **BRAF V600 status** | |
| Mutated | 40 (26.5) |
| Wild type | 46 (30.5) |
| Unknown/indetermined | 65 (43.0) |
| **SLNB** |  |
| Positive | 34 (22.5) |
| Negative | 80 (53.0) |
| Not done | 37 (24.5) |
| **Number of positive lymph nodes** |  |
| 0 | 81 (53.7) |
| 1 | 18 (11.9) |
| 2 | 7 (4.6) |
| ≥3 | 9 (5.9) |
| Unknown/Not done | 36 (23.9) |
| **Neurotropism** | |
| Absent | 97 (64.3) |
| Present | 2 (1.3) |
| Unknown | 52 (34.4) |
| **Lymphovascular invasion** | |
| Absent | 91 (60.3) |
| Present | 9 (6.0) |
| Unknown | 51 (33.7) |
| **Satellitosis and in-transit metastases** | |
| Multiple types | 1 (0.7) |
| Satellites | 1 (0.7) |
| None | 149 (98.6) |
| **Mitotic rate** | |
| High | 35 (23.2) |
| Intermediate | 48 (31.8) |
| Low | 14 (9.3) |
| Unknown | 54 (35.7) |
| **Total number of comorbidities** | |
| 0 | 108 (71.5) |
| 1 | 25 (16.6) |
| 2 | 8 (5.3) |
| 3 | 6 (4.0) |
| >3 | 4 (2.6) |
| **Year of diagnosis** |  |
| 2020 | 10 (6.6) |
| 2020/2021 | 7 (4.6) |
| 2021 | 30 (19.9) |
| 2021/2022 | 3 (2.0) |
| 2022 | 24 (15.9) |
| 2022/2023 | 6 (4.0) |
| 2023 | 38 (25.2) |
| 2023/2024 | 4 (2.6) |
| 2024 | 29 (19.2) |
| **MDT treatment recommendation** | |
| Surgery alone | 102 (67.6) |
| Surgery + adjuvant therapy | 5 (3.3) |
| Immunotherapy | 20 (13.2) |
| Immunotherapy + radiotherapy | 4 (2.6) |
| BRAF/MEK | 7 (4.6) |
| BRAF/MEK + radiotherapy | 6 (4.0) |
| Supportive care | 6 (4.0) |
| Radiotherapy + supportive care | 1 (0.7) |
| **Number of MDT treatment options** | |
| 1 | 69 (45.7) |
| 2 | 42 (27.8) |
| ≥3 | 40 (26.5) |

**Supplementary Table 2. Post-hoc pairwise comparisons between LLMs across evaluation domains using Dunn’s test with Bonferroni correction**

| **Domain** | **LLM** | **LLM** | **Z** | **p-value (Bonferroni)** | **Significant** |
| --- | --- | --- | --- | --- | --- |
| Clarity & Coherence | ChatGPT-4o | ChatGPT-5 Thinking | 4.766 | p < 0.001 | **** |
|  | ChatGPT-4o | Gemini 2.5 Pro | -1.397 | 0.975 | ns |
|  | ChatGPT-4o | DeepSeek-V3.2 | -5.551 | p < 0.001 | **** |
|  | ChatGPT-5 Thinking | Gemini 2.5 Pro | -6.163 | p < 0.001 | **** |
|  | ChatGPT-5 Thinking | DeepSeek-V3.2 | -10.317 | p < 0.001 | **** |
|  | Gemini 2.5 Pro | DeepSeek-V3.2 | -4.154 | p < 0.001 | *** |
| Clinical Applicability | ChatGPT-4o | ChatGPT-5 Thinking | 1.636 | 0.611 | ns |
|  | ChatGPT-4o | Gemini 2.5 Pro | -4.774 | p < 0.001 | **** |
|  | ChatGPT-4o | DeepSeek-V3.2 | -6.531 | p < 0.001 | **** |
|  | ChatGPT-5 Thinking | Gemini 2.5 Pro | -6.410 | p < 0.001 | **** |
|  | ChatGPT-5 Thinking | DeepSeek-V3.2 | -8.167 | p < 0.001 | **** |
|  | Gemini 2.5 Pro | DeepSeek-V3.2 | -1.757 | 0.474 | ns |
| Coverage | ChatGPT-4o | ChatGPT-5 Thinking | 9.304 | p < 0.001 | **** |
|  | ChatGPT-4o | Gemini 2.5 Pro | 1.801 | 0.43 | ns |
|  | ChatGPT-4o | DeepSeek-V3.2 | -0.046 | 1 | ns |
|  | ChatGPT-5 Thinking | Gemini 2.5 Pro | -7.502 | p < 0.001 | **** |
|  | ChatGPT-5 Thinking | DeepSeek-V3.2 | -9.349 | p < 0.001 | **** |
|  | Gemini 2.5 Pro | DeepSeek-V3.2 | -1.847 | 0.389 | ns |
| Explanation and Support with Evidence | ChatGPT-4o | ChatGPT-5 Thinking | 14.310 | p < 0.001 | **** |
|  | ChatGPT-4o | Gemini 2.5 Pro | 8.843 | p < 0.001 | **** |
|  | ChatGPT-4o | DeepSeek-V3.2 | 7.182 | p < 0.001 | **** |
|  | ChatGPT-5 Thinking | Gemini 2.5 Pro | -5.467 | p < 0.001 | **** |
|  | ChatGPT-5 Thinking | DeepSeek-V3.2 | -7.128 | p < 0.001 | **** |
|  | Gemini 2.5 Pro | DeepSeek-V3.2 | -1.661 | 0.58 | ns |
| Guideline concordance | ChatGPT-4o | ChatGPT-5 Thinking | 4.576 | p < 0.001 | **** |
|  | ChatGPT-4o | Gemini 2.5 Pro | -2.043 | 0.246 | ns |
|  | ChatGPT-4o | DeepSeek-V3.2 | -2.985 | 0.017 | * |
|  | ChatGPT-5 Thinking | Gemini 2.5 Pro | -6.619 | p < 0.001 | **** |
|  | ChatGPT-5 Thinking | DeepSeek-V3.2 | -7.561 | p < 0.001 | **** |
|  | Gemini 2.5 Pro | DeepSeek-V3.2 | -0.942 | 1 | ns |

**Supplementary Table 3. Subgroup analysis of agreement between LLMs and MDT treatment recommendations. Results are presented as mean Likert scores (1–4) and standard deviation (SD).**

| **Variable** | **Category** |  | **ChatGPT-4o** | | **ChatGPT-5 Thinking** | | **Gemini 2.5 Pro** | | **DeepSeek-V3.2** | |
| --- | --- | --- | --- | --- | --- | --- | --- | --- | --- | --- |
|  |  | **N** | **Mean** | **SD** | **Mean** | **SD** | **Mean** | **SD** | **Mean** | **SD** |
| Sex | F | 62 | 3.32 | 0.41 | 3.32 | 0.36 | 3.15 | 0.36 | 3.03 | 0.41 |
|  | M | 89 | 3.21 | 0.41 | 3.28 | 0.35 | 3.13 | 0.41 | 3.04 | 0.36 |
| ECOG | 0-1 | 140 | 3.26 | 0.41 | 3.30 | 0.34 | 3.13 | 0.39 | 3.04 | 0.38 |
|  | 2 | 4 | 2.88 | 0.25 | 3.06 | 0.31 | 3.25 | 0.65 | 3.19 | 0.31 |
|  | 3 | 7 | 3.46 | 0.27 | 3.32 | 0.57 | 3.21 | 0.34 | 3.04 | 0.53 |
| AJCC stage at presentation | 0 | 9 | 3.42 | 0.54 | 3.50 | 0.47 | 3.22 | 0.36 | 3.22 | 0.36 |
|  | IA | 10 | 3.50 | 0.35 | 3.55 | 0.33 | 3.45 | 0.39 | 3.17 | 0.43 |
|  | IB | 20 | 3.34 | 0.60 | 3.34 | 0.30 | 3.20 | 0.34 | 3.08 | 0.39 |
|  | IIA | 14 | 3.48 | 0.36 | 3.29 | 0.46 | 3.11 | 0.40 | 3.04 | 0.45 |
|  | IIB | 27 | 3.24 | 0.28 | 3.34 | 0.35 | 3.04 | 0.45 | 3.04 | 0.40 |
|  | IIC | 16 | 3.09 | 0.34 | 3.16 | 0.29 | 3.02 | 0.50 | 2.88 | 0.37 |
|  | IIIB | 1 | 2.75 | NA | 2.75 | NA | 3.25 | NA | 2.25 | NA |
|  | IIIC | 25 | 3.07 | 0.43 | 3.14 | 0.28 | 3.01 | 0.31 | 2.94 | 0.37 |
|  | IIID | 2 | 3.25 | 0.35 | 3.75 | 0.35 | 3.38 | 0.18 | 3.38 | 0.18 |
|  | IV | 27 | 3.24 | 0.25 | 3.29 | 0.30 | 3.21 | 0.31 | 3.10 | 0.28 |
| Primary tumor site | Head/Neck | 33 | 3.29 | 0.41 | 3.36 | 0.34 | 3.11 | 0.39 | 3.12 | 0.41 |
|  | Lower extremity | 25 | 3.25 | 0.45 | 3.28 | 0.38 | 3.13 | 0.39 | 2.97 | 0.42 |
|  | Trunk | 62 | 3.23 | 0.38 | 3.27 | 0.39 | 3.14 | 0.38 | 3.01 | 0.39 |
|  | Upper extremity | 19 | 3.26 | 0.54 | 3.32 | 0.27 | 3.13 | 0.50 | 3.08 | 0.33 |
| Histologic subtype | Desmoplastic melanoma | 1 | 3.50 | NA | 4.00 | NA | 3.00 | NA | 3.50 | NA |
|  | Lentigo maligna melanoma | 2 | 3.00 | 0.35 | 3.25 | 0.00 | 2.88 | 0.53 | 2.88 | 0.53 |
|  | Nodular melanoma | 60 | 3.20 | 0.47 | 3.28 | 0.36 | 3.01 | 0.43 | 2.95 | 0.38 |
|  | Not otherwise specified | 71 | 3.28 | 0.36 | 3.30 | 0.34 | 3.21 | 0.35 | 3.08 | 0.36 |
|  | Spitzoid melanoma | 15 | 3.30 | 0.33 | 3.30 | 0.41 | 3.23 | 0.26 | 3.15 | 0.43 |
|  | Superficial spreading melanoma | 2 | 4.00 | 0.00 | 3.62 | 0.18 | 3.62 | 0.18 | 3.38 | 0.18 |
| BRAF V600 status | Unknown | 64 | 3.38 | 0.40 | 3.37 | 0.38 | 3.18 | 0.40 | 3.12 | 0.36 |
|  | Mutated | 40 | 3.22 | 0.36 | 3.26 | 0.36 | 3.14 | 0.34 | 3.02 | 0.36 |
|  | Wild type | 46 | 3.12 | 0.43 | 3.25 | 0.29 | 3.05 | 0.42 | 2.95 | 0.41 |
|  | Indetermined | 1 | 3.25 | NA | 2.75 | NA | 3.25 | NA | 2.75 | NA |
| Ulceration | Absent | 56 | 3.30 | 0.47 | 3.30 | 0.40 | 3.18 | 0.38 | 3.03 | 0.43 |
|  | Not applicable | 10 | 3.22 | 0.25 | 3.38 | 0.44 | 3.40 | 0.32 | 3.15 | 0.27 |
|  | Present | 85 | 3.23 | 0.38 | 3.29 | 0.31 | 3.07 | 0.39 | 3.03 | 0.36 |
| Breslow thickness (mm) | / | 8 | 3.19 | 0.26 | 3.34 | 0.33 | 3.31 | 0.35 | 3.19 | 0.26 |
|  | 0 | 8 | 3.44 | 0.58 | 3.47 | 0.49 | 3.25 | 0.38 | 3.19 | 0.37 |
|  | 0.8-1.0 | 10 | 3.48 | 0.36 | 3.42 | 0.26 | 3.35 | 0.34 | 3.28 | 0.40 |
|  | 1.0-2.0 | 23 | 3.40 | 0.56 | 3.36 | 0.38 | 3.11 | 0.43 | 2.93 | 0.39 |
|  | 2.0-4.0 | 37 | 3.23 | 0.32 | 3.24 | 0.31 | 3.07 | 0.39 | 3.01 | 0.41 |
|  | <0.8 | 4 | 3.62 | 0.25 | 3.69 | 0.38 | 3.50 | 0.00 | 3.31 | 0.38 |
|  | >4.0 | 61 | 3.14 | 0.36 | 3.23 | 0.34 | 3.08 | 0.38 | 3.00 | 0.35 |
| SLNB | Not done | 37 | 3.34 | 0.38 | 3.37 | 0.41 | 3.31 | 0.30 | 3.18 | 0.35 |
|  | Negative | 80 | 3.26 | 0.43 | 3.30 | 0.33 | 3.07 | 0.42 | 2.98 | 0.39 |
|  | Positive | 34 | 3.16 | 0.39 | 3.22 | 0.34 | 3.10 | 0.36 | 3.03 | 0.37 |
| Number of positive lymph nodes | / | 36 | 3.34 | 0.38 | 3.38 | 0.42 | 3.31 | 0.31 | 3.17 | 0.35 |
|  | 0 | 81 | 3.26 | 0.43 | 3.30 | 0.33 | 3.07 | 0.42 | 2.98 | 0.39 |
|  | 1 | 18 | 3.15 | 0.45 | 3.24 | 0.29 | 3.15 | 0.35 | 3.00 | 0.37 |
|  | 2 | 7 | 3.07 | 0.28 | 3.00 | 0.25 | 2.96 | 0.34 | 3.00 | 0.38 |
|  | 3 | 4 | 2.94 | 0.24 | 3.06 | 0.24 | 3.06 | 0.43 | 3.06 | 0.24 |
|  | 4 | 1 | 3.50 | NA | 3.50 | NA | 3.50 | NA | 3.50 | NA |
|  | 6 | 2 | 3.62 | 0.18 | 3.62 | 0.53 | 3.25 | 0.35 | 3.38 | 0.18 |
|  | 7 | 2 | 3.38 | 0.18 | 3.62 | 0.53 | 2.88 | 0.53 | 2.75 | 0.71 |
| Neurotropism | Unknown | 52 | 3.23 | 0.40 | 3.30 | 0.35 | 3.15 | 0.41 | 3.02 | 0.39 |
|  | Absent | 97 | 3.28 | 0.42 | 3.29 | 0.36 | 3.12 | 0.38 | 3.04 | 0.38 |
|  | Present | 2 | 3.12 | 0.18 | 3.38 | 0.18 | 3.12 | 0.18 | 3.25 | 0.00 |
| Lymphovascular invasion | Unknown | 51 | 3.23 | 0.40 | 3.29 | 0.34 | 3.15 | 0.42 | 3.02 | 0.39 |
|  | Absent | 91 | 3.28 | 0.43 | 3.30 | 0.35 | 3.12 | 0.38 | 3.05 | 0.38 |
|  | Present | 9 | 3.17 | 0.33 | 3.33 | 0.47 | 3.22 | 0.42 | 3.06 | 0.37 |
| Satellitosis and in-transit metastases | Multiple types | 1 | 2.50 | NA | 3.00 | NA | 3.00 | NA | 3.25 | NA |
|  | Satellites | 1 | 4.00 | NA | 3.50 | NA | 3.25 | NA | 3.50 | NA |
|  | None | 149 | 3.26 | 0.40 | 3.30 | 0.35 | 3.13 | 0.39 | 3.04 | 0.38 |
| Mitotic rate | Unknown | 54 | 3.22 | 0.41 | 3.30 | 0.36 | 3.15 | 0.41 | 3.02 | 0.38 |
|  | High | 35 | 3.29 | 0.32 | 3.29 | 0.32 | 3.09 | 0.39 | 3.07 | 0.32 |
|  | Intermediate | 48 | 3.21 | 0.46 | 3.24 | 0.37 | 3.10 | 0.39 | 2.97 | 0.40 |
|  | Low | 14 | 3.48 | 0.42 | 3.52 | 0.29 | 3.29 | 0.31 | 3.29 | 0.37 |
| Number of comorbidities | 0 | 108 | 3.26 | 0.42 | 3.31 | 0.35 | 3.12 | 0.40 | 3.05 | 0.37 |
|  | 1 | 25 | 3.19 | 0.41 | 3.28 | 0.40 | 3.21 | 0.40 | 2.90 | 0.44 |
|  | 2 | 8 | 3.25 | 0.33 | 3.12 | 0.42 | 3.09 | 0.30 | 3.06 | 0.37 |
|  | 3 | 6 | 3.33 | 0.49 | 3.33 | 0.30 | 3.17 | 0.30 | 3.21 | 0.33 |
|  | >3 | 4 | 3.38 | 0.48 | 3.25 | 0.20 | 3.06 | 0.55 | 3.31 | 0.12 |
| MDT - treatment  recommendation | BRAF/MEK | 7 | 3.21 | 0.27 | 3.29 | 0.37 | 3.14 | 0.32 | 3.00 | 0.38 |
|  | BRAF/MEK + radiotherapy | 6 | 3.21 | 0.25 | 3.25 | 0.16 | 3.17 | 0.26 | 3.21 | 0.10 |
|  | Immunotherapy | 20 | 3.24 | 0.32 | 3.28 | 0.34 | 3.28 | 0.33 | 3.10 | 0.30 |
|  | Immunotherapy + radiotherapy | 4 | 3.12 | 0.32 | 3.19 | 0.31 | 3.19 | 0.47 | 3.25 | 0.20 |
|  | Supportive care | 6 | 3.42 | 0.34 | 3.25 | 0.59 | 3.17 | 0.30 | 2.96 | 0.53 |
|  | Radiotherapy + supportive care | 1 | 3.25 | NA | 2.75 | NA | 3.75 | NA | 2.75 | NA |
|  | Surgery + adjuvant therapy | 5 | 3.25 | 0.35 | 3.35 | 0.22 | 3.20 | 0.21 | 3.25 | 0.00 |
|  | Surgery alone | 102 | 3.26 | 0.46 | 3.32 | 0.36 | 3.09 | 0.42 | 3.01 | 0.41 |
| Number of MDT treatment options | 1 | 69 | 3.39 | 0.44 | 3.40 | 0.35 | 3.21 | 0.43 | 3.11 | 0.38 |
|  | 2 | 42 | 3.05 | 0.38 | 3.15 | 0.34 | 2.94 | 0.36 | 2.89 | 0.42 |
|  | ≥3 | 40 | 3.23 | 0.29 | 3.28 | 0.31 | 3.21 | 0.28 | 3.08 | 0.30 |

NA: Not applicable
